# Swept-source OCT findings in shaken baby syndrome: case report

**DOI:** 10.1186/s12886-020-01666-9

**Published:** 2020-10-07

**Authors:** Imen Ksiaa, Mohamed Ghachem, Habib Besbes, Sana Khochtali, Slaheddine Chouchane, Moncef Khairallah

**Affiliations:** 1 Department of Ophthalmology, Faculty of Medicine, Fattouma Bourguiba University Hospital, University of Monastir, Monastir, Tunisia; 2 Department of Pediatrics, Faculty of Medicine, Fattouma Bourguiba University Hospital, University of Monastir, Monastir, Tunisia

**Keywords:** Shaken baby syndrome, Swept source optical coherence tomography, Retinal hemorrhages, Vitreoretinal interface, Case report

## Abstract

**Background:**

Our purpose was to document the swept source optical coherence tomography (SSOCT) findings in a patient with Shaken baby syndrome (SBS).

**Case presentation:**

SSOCT was obtained without sedation in a six-month-old girl with bilateral multilayered retinal hemorrhages due to SBS. It documented vitreoretinal interface abnormalities, including internal limiting membrane (ILM) detachment with retinal traction, in association with other specific changes in the inner and outer retinal layers. Six weeks later, retinal hemorrhages had substantially resolved, and there was optic disc pallor. OCT showed ILM reattachment with release of retinal traction and the development of severe diffuse retinal atrophy involving the fovea.

**Conclusions:**

SS OCT can provide useful information in SBS, revealing a wide variety of vitreoretinal interface, inner, and outer retinal changes not detected by clinical examination. It also may have a prognostic value over follow-up.

## Background

Shaken baby syndrome (SBS), also known as abusive head trauma, refers to a constellation of clinical findings including bilateral retinal hemorrhages, subdural hemorrhage, and anoxic encephalopathy [1]. Retinal hemorrhages occur in approximately 85% of cases and they typically are numerous, multilayered and widespread, involving the posterior pole and periphery [2]. Optical coherence tomography (OCT) has been found to be useful in the evaluation of retinal hemorrhages and in the detection and characterization of associated vitreoretinal abnormalities in patients with SBS. However, there are only very few reports on the use of SD-OCT, and data on the newly-introduced swept-source (SS) OCT are lacking [2–5]. We herein report a case of SBS documented with SS OCT.

## Case presentation

**Fig. 1:**
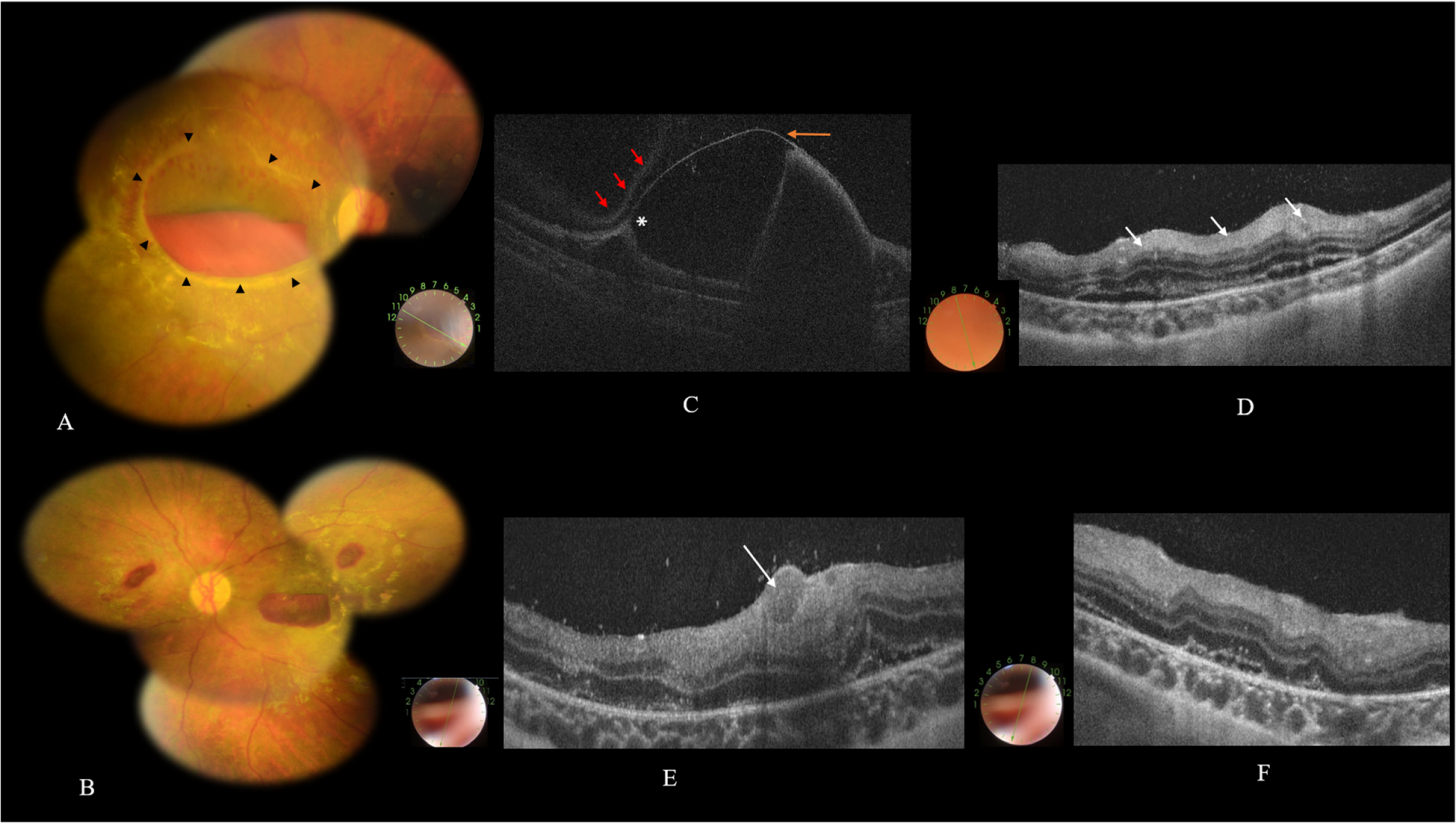
**a**, **b** Fundus photography showing bilateral intraretinal and preretinal hemorrhages, with a boat-shaped premacular hematoma, which is in the right eye larger in size and surrounded with an elevated ring-shaped white retinal fold (arrowheads). **c** SS OCT scan through the macula of the right eye showing a dome-shaped highly reflective band corresponding to a detached internal limiting membrane (ILM) (orange arrow) with associated posterior shadowing from dense sub-ILM hemorrhage. Note the presence of retinal traction at the upper edge of the detached ILM corresponding to the perimacular elevated retinal fold seen clinically (asterix). Note also the fainter reflecting posterior hyaloid overlying the detached ILM superiorly (red arrows). **d** SS OCT scan through nasal retina showing diffuse inner retinal hyperreflectivity (arrows) and multifocal serous retinal detachment. **e**, **f** SS OCT scans of the left eye showing hyperreflective vitreous dots, a sub-ILM hemorrhage (arrow), wave-shaped retinal layers deformation, diffuse inner retinal hyperreflectivity, ellipsoid zone disruption, intraretinal hyperreflective dots, and serous retinal detachment

A previously healthy six-month-old girl was brought to the emergency department for paroxysmal crying with brief episodes of loss of consciousness. On physical examination there was a bulging fontanel with associated weak axial posture. A cerebral computed tomography (CT)-scan demonstrated extensive bilateral subdural hemorrhages. The child was admitted to the Pediatric Intensive Care Unit. Ophthalmological examination revealed a poor pupillary response to bright light in both eyes. There were no external signs related to ocular trauma. Fundus examination by indirect ophthalmoscopy revealed bilateral preretinal and intraretinal hemorrhages involving the posterior pole and midperiphery. There was a bilateral boat-shaped premacular hemorrhage. This hemorrhage was larger and associated with a prominent surrounding ring-shaped white retinal fold in the right eye (Fig. 1). A diagnosis of SBS was made based on the patient’s neurological status, and fundoscopic and CT-scan findings. The baby-sitter looking after the child confessed to abusing her. The neurological condition was managed with intravenous mannitol, along with close monitoring.

Three days after hospitalization, the patient underwent swept source OCT imaging with the DRI OCT Triton plus (Topcon, Tokyo, Japan). Multiple SS-OCT scans could be obtained without sedation, the infant being held and her eyelids kept open by an assistant. OCT confirmed multilayered retinal hemorrhages in both eyes. It showed a dome-shaped detachment of the internal limiting membrane (ILM) overlying the macular hematoma bilaterally, with associated perifoveal retinal traction corresponding to the retinal fold seen clinically in the right eye (Fig. 1). Other SS OCT findings included hyperreflective vitreous dots representing individual red blood cells, wave-shaped retinal layers deformation, diffuse inner retinal hyperreflectivity, ellipsoid zone disruption, intraretinal hyperreflective dots, and serous retinal detachment. The foveal pit was not identifiable. The retinal pigment epithelium and choroid appeared to be normal in both eyes.

**Fig. 2:**
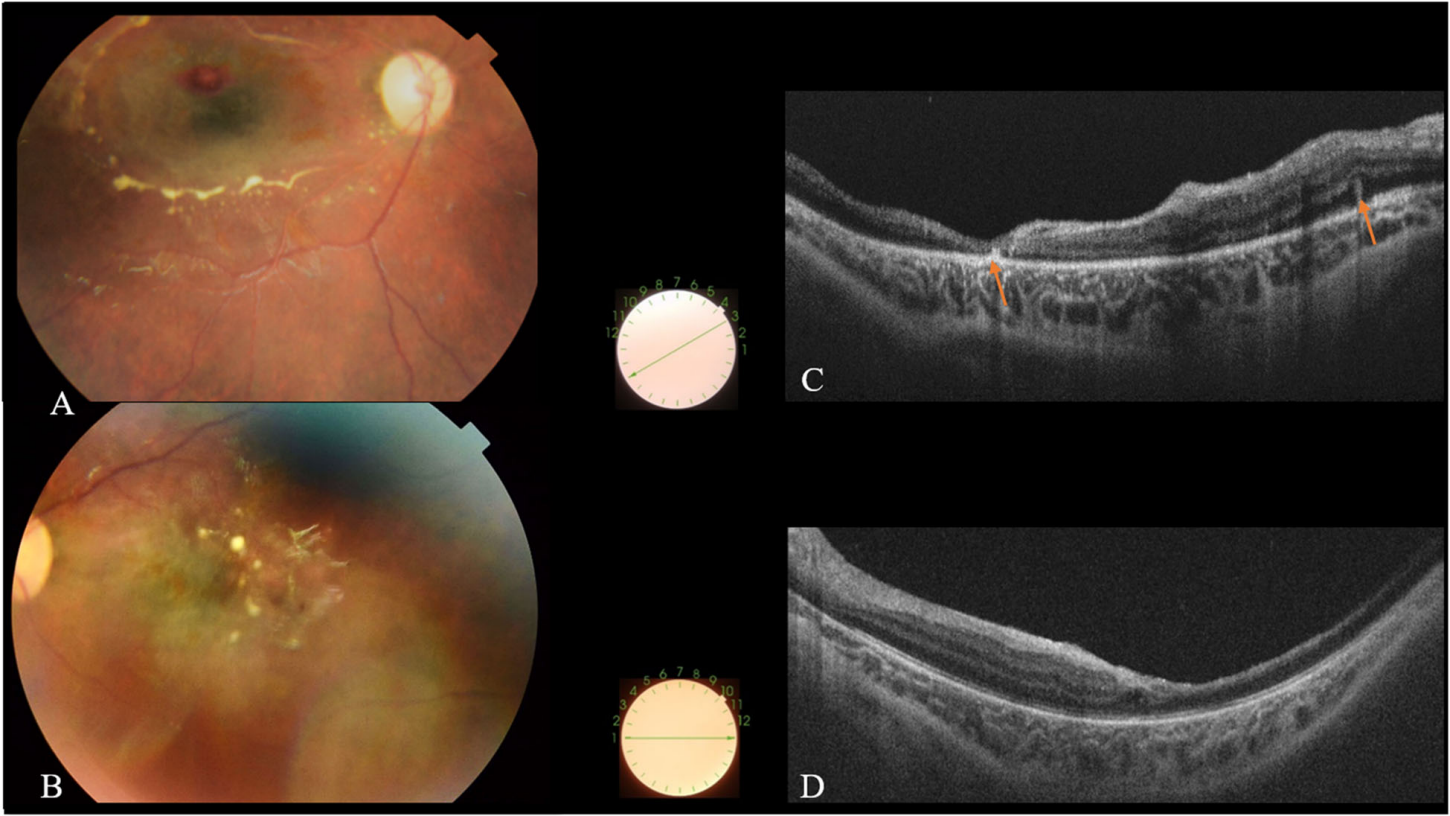
**a**, **b** Fundus photographs taken 6 weeks after initial presentation showing a substantial resolution of retinal and preretinal hemorrhages, multiple areas of residual subretinal fibrosis, and optic disc pallor, mainly in the right eye. **c**, **d** SS OCT scans showing complete reattachment of the detached ILM with release of retinal traction in the right eye, a marked temporal retinal atrophy involving the fovea, and subretinal hyperreflective lesions (arrows), corresponding to the areas of subretinal fibrosis seen clinically

Sequential follow-up examinations showed gradual resolution of the neurological symptoms and improvement of pupillary response to light. Six weeks after initial examination, retinal and preretinal hemorrhages had substantially resolved, and there were bilateral areas of subretinal fibrosis and optic disc pallor, mainly in the right eye (Fig. 2).

SS OCT, six weeks after initial presentation, showed complete reattachment of the detached ILM, with release of retinal traction and resolution of other acute findings. There was a bilateral marked diffuse retinal atrophy involving the fovea with associated subretinal hyperreflective lesions corresponding to the areas of subretinal fibrosis seen clinically (Fig. 2).

## Discussion and conclusions

To the best of our knowledge, this report is the first to describe the use of SS OCT in the assessment and monitoring of retinal disease associated with SBS. Thanks to the faster acquisition times of SSOCT technology, OCT scans could be obtained without sedation, although an assistant was required to hold the infant’s head. Until recently, time domain and conventional or hand-held spectral domain (SD)-OCT have been rarely used in very young children with acute SBS [2–5]. OCT imaging usually was performed under sedation or even general anesthesia, and OCT follow-up data were lacking in most cases. The anatomic location of preretinal hemorrhages in SBS is usually described as subhyaloid in type [2–5]. Our SS OCT findings provide evidence of sub-ILM location of premacular hematoma similar to that previously described in Valsalva retinopathy [6]. Our results, consistent with previous data on SD-OCT, [2, 3, 5] show evidence of vitreoretinal interface pathology in association with multilayered hemorrhages in SBS. OCT findings may include focal posterior vitreous detachment, retinal traction, perimacular folds, retinoschisis, disinsertion of the ILM, epiretinal membrane, and macular hole. These data led the theory of vitreoretinal traction due to shearing forces induced by shaking to be the most widely accepted hypothesis on the pathogenesis of retinal disease associated with SBS [2, 5, 7, 8]. Alternatively, increased intracranial pressure due to extradural hemorrhages could be a potential mechanism of intraocular bleeding in our patient. Other hyoptheses include increased intrathoracic pressure leading to sudden raise of retinal venous pressure, and retinal hypoxia [7, 8].

Another dimension of ocular changes in SBS was described in the field of forensic pathology. OCT findings may provide valuable information suggestive of child abuse in the absence of external evidence of trauma [9].

Our study expands the OCT spectrum of SBS to include a wide variety of previously undescribed vitreous and retinal changes, including alterations of the outer retinal layers. On follow-up OCT examination, severe retinal atrophic changes became evident after resolution of hemorrhages and other acute findings. Our data show that macular atrophy may be an important causative mechanism of severe vision loss in children with SBS.

In conclusion, thanks to its faster acquisition time and deeper penetration, SSOCT may be useful in the evaluation and monitoring of ocular disease in awake young children with SBS. It can provide useful information, revealing a wide variety of vitreoretinal interface, inner, and outer retinal changes not detected by clinical examination. It also may have a prognostic value over follow-up.

## Data Availability

Not applicable.

## Abbreviations

CT: Computed tomography
ILM: Internal limiting membrane
SBS: Shaken baby syndrome
SD: Spectral domain
SSOCT: Swept source optical coherence tomography
